# Supplementary material for: Molecular mechanism of complement interference by Streptococcus pyogenes nuclease A
Source: Commun Biol. 2026 Sep 23;9:1245. doi: 10.1038/s42003-026-10956-9 (PMC13601614; doi:10.1038/s42003-026-10956-9)
Supplement: Supplementary file 1 [file 42003_2026_10956_MOESM1_ESM.pdf]

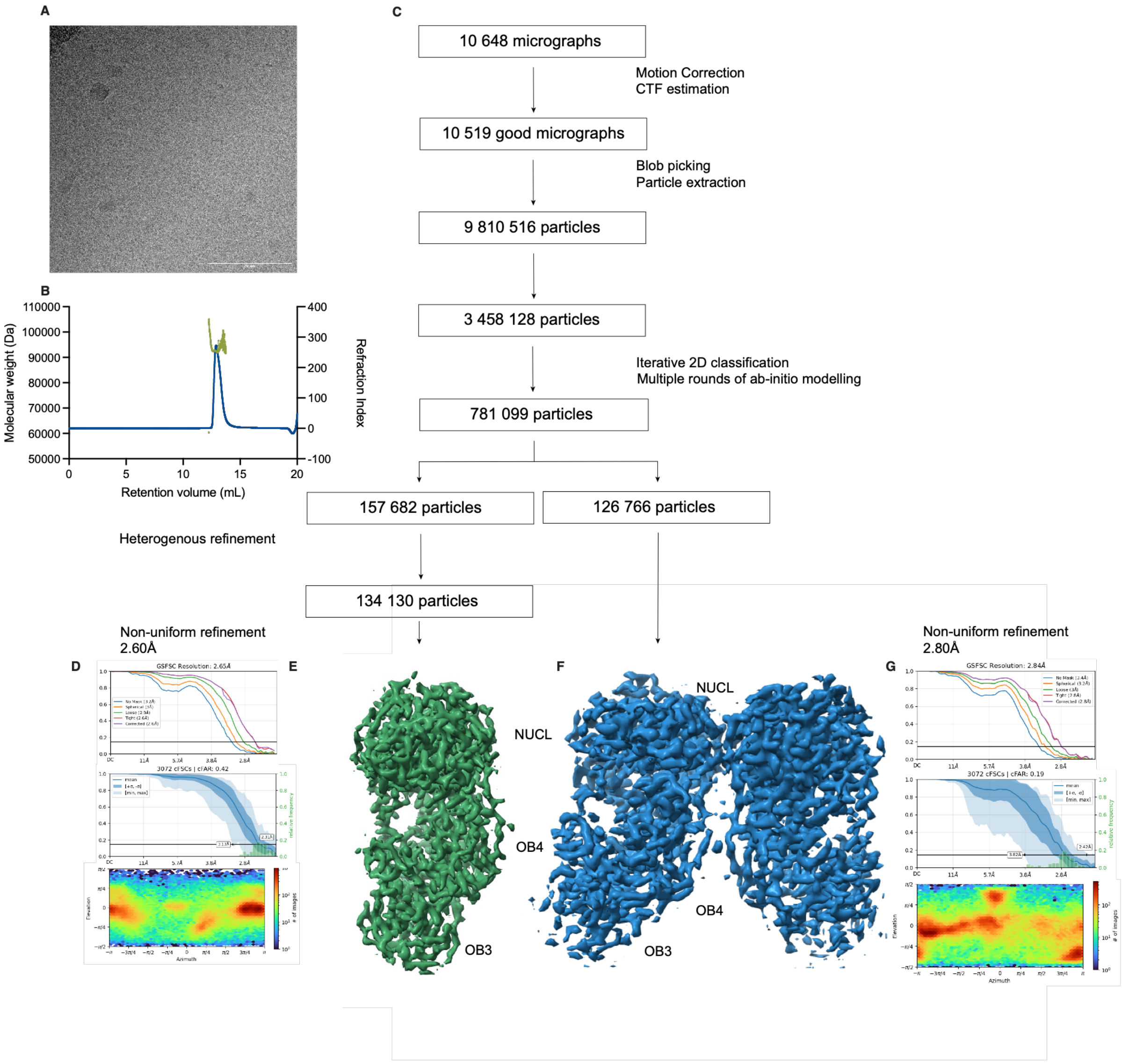

Supplementary figure 1. Overview of cryoEM data processing. A) Micrograph of SpnA at a nominal magnification of 215000x (scalebar of 20 nm). **B)** SEC-MALS data of SpnA, showing molecular weight (left-side y-axis, green), retention volume in ml (x-axis) and refraction index (right-hand y-axis). **C)** Flowchart showing the image processing workflow for the SpnA cryoEM dataset. In total, 10 649 micrographs were collected on a Titan Krios 300 kV microscope equipped with a Falcon 4i direct electron detector and processed using cryoSPARC. Out of an original of 9 810 000 extracted particles, **D-E)** approximately 134 000 contributed the monomeric structure of SpnA at a resolution of 2.60 Å, and **F-G)** around 127 000 to the dimeric structure at 2.80 Å resolution. The graphs for the GSFSC curve (top), cFAR (middle) and viewing direction distribution plot (bottom) are indicated for the monomer and dimer in **D)** and **G)**. The oligonucleotide-binding domains (OB 3-4) and endo/exonuclease domain (NUCL) are indicated in **E)** and **F).**

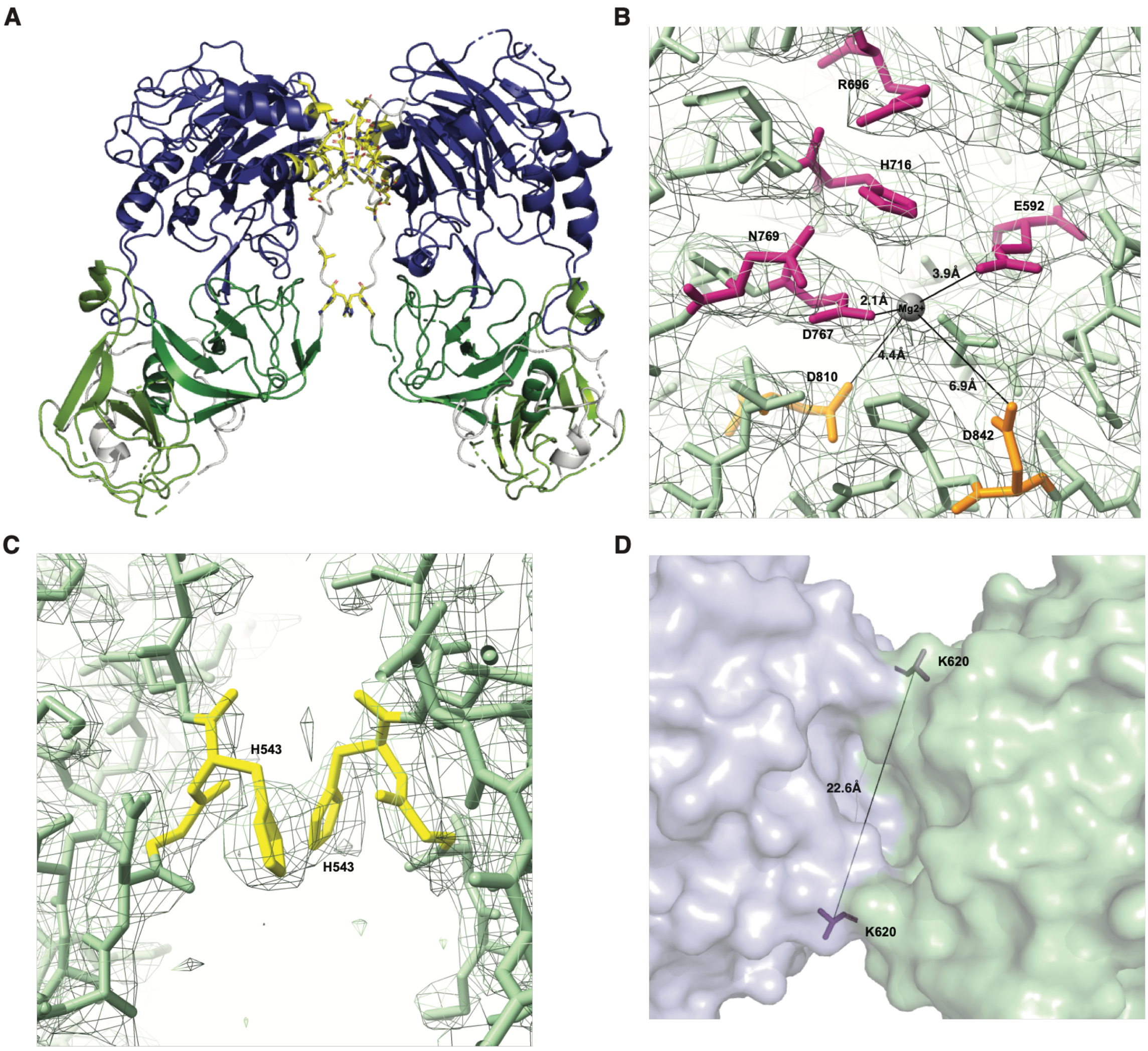

Supplementary figure 2: Dimeric structure of SpnA. **A)** CryoEM structure of dimeric SpnA at 2.80Å. The endo/exonuclease domain (NUCL) is indicated in dark blue, the oligonucleotide-binding domain (OB) 4 in dark green and OB3 in light green. The dimerization interfaces determined by SpotOn [36] are highlighted in yellow, with a close-up of H543-H543 in panel **B**. **B)** Zoom-in of the dimerization interface determined by SpotOn between H543 (chain A) and H543 (chain B). The EM map is shown at a threshold of 0.15 (green). **C**) The active site with the residues involved in catalysis. The suggested active site residues [38] are indicated in pink. According to Chalmers *et al*. [38], R696 and N769 bind the nucleic acid substrate, H716, D767 and D810 are essential for catalysis, and E592 coordinate the Mg^{2+} -ion. Distances in the active site to the Mg^{2+} -ion are indicated. **D)** The dimerization interface is supported by an interchain cross-link between lysine (K620) on chain A (blue) and K620 on chain B (green).

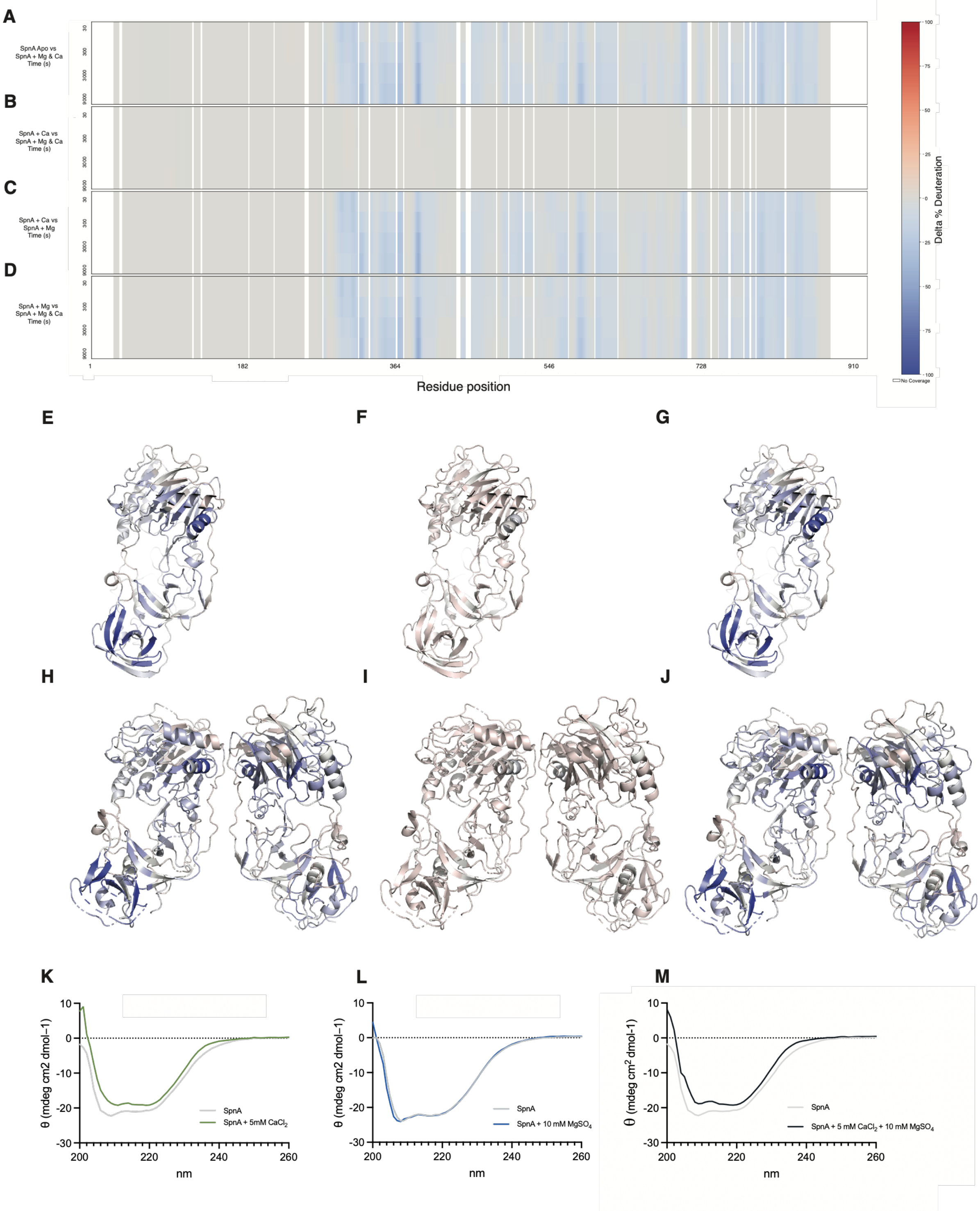

Supplementary figure 3: The structure of SpnA is stabilised in the presence of Ca^{2+} - but not Mg^{2+} - ions. HDX-MS deuterium uptake plots (delta % deuteration). **A)** SpnA apo compared to SpnA in presence of Mg^{2+}- and Ca^{2+} -ions; **B)** SpnA in presence of Ca^{2} -ions compared to SpnA in presence of Mg^{2+}- and Ca^{2+} -ions; **C)** SpnA in presence of Ca^{2+} -ions compared to SpnA in presence of Mg^{2+}-ions; and **D)** SpnA in presence of Mg^{2+} -ions compared to SpnA in presence of Mg^{2+}- and Ca^{2+} -ions. The data is shown at time points 30, 300, 3000 and 9000s. The deuterium uptake propensity (delta % deuteration) is shown to the right. The cartoon representation of the monomeric and dimeric SpnA structures are colored according to

the delta % deuteration signal acquired from HDX-MS data. **E**) Monomeric SpnA (PDB: 9SF8) overlaid with SpnA (apo) vs. SpnA in the presence of Ca^{2+}-ions; **F**) overlaid with SpnA (apo) vs. SpnA in the presence of Mg^{2+}-ions; and **G**) overlaid with SpnA (apo) vs. SpnA in the presence of Mg^{2+} - and Ca^{2+}-ions. **H**) Dimeric SpnA (PDB: 9SF9) overlaid with SpnA (apo) vs. SpnA in the presence of Ca^{2+}-ions; **I**) overlaid with SpnA (apo) vs. SpnA in the presence of Mg^{2+}-ions; and **J**) overlaid with SpnA (apo) vs. SpnA in the presence of Mg^{2+} - and Ca^{2+}-ions. Panels E-J were generated in pyMOL. **K)** CD spectroscopy data of SpnA alone and in the presence of 5 mM $CaCl_2$, **L)** SpnA alone and in the presence of 10 mM $MgSO_4$, and **M)** SpnA alone and in the presence of 10 mM $MgSO_4$ and 5 mM $CaCl_2$. Panels **K-M)** indicate a conformational shift in SpnA in the presence of $CaCl_2$, but not with $MgSO_4$ alone.

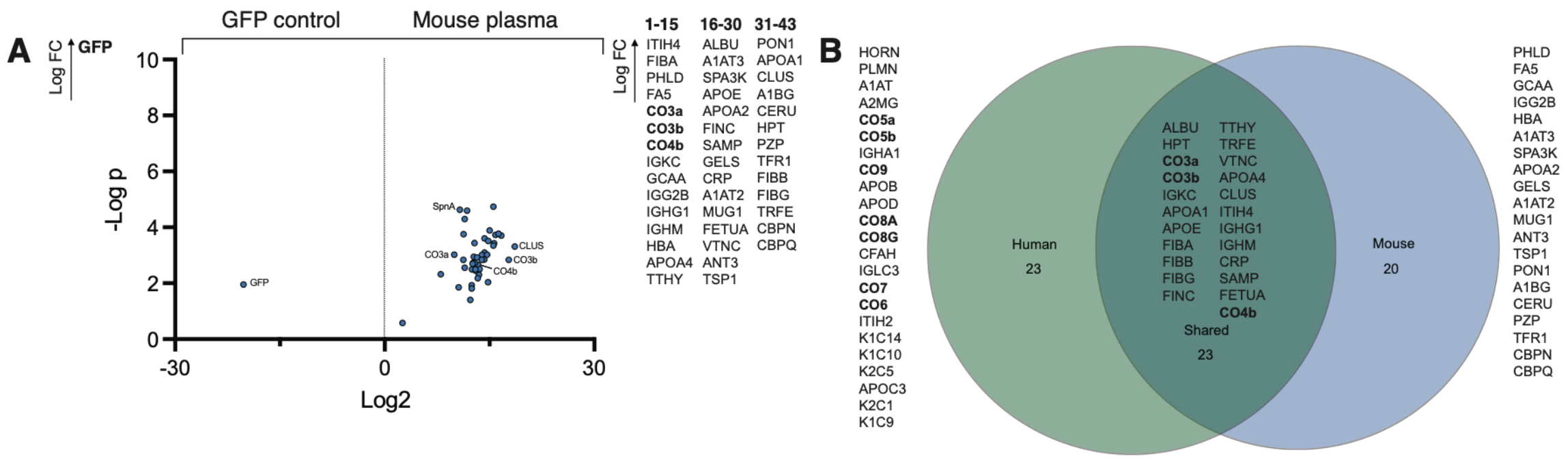

Supplementary figure 4. Mouse plasma interactome of SpnA. **A**) Volcano plot comparing the mouse plasma interactome of SpnA to GFP. Significantly enriched proteins are indicated on the sides of the plot in Log2 foldchange (FC) order. **B**) Venn diagram comparing the significantly enriched human and mouse SpnA interactors, revealing a core, species independent interactome of 22 proteins, whereas 22 proteins – including the complement system MAC – are human specific, and 20 are mouse specific. The Venn diagram is generated using BioVenn [59]. For the AP-MS assays, n = 5 independent pulldowns were performed.

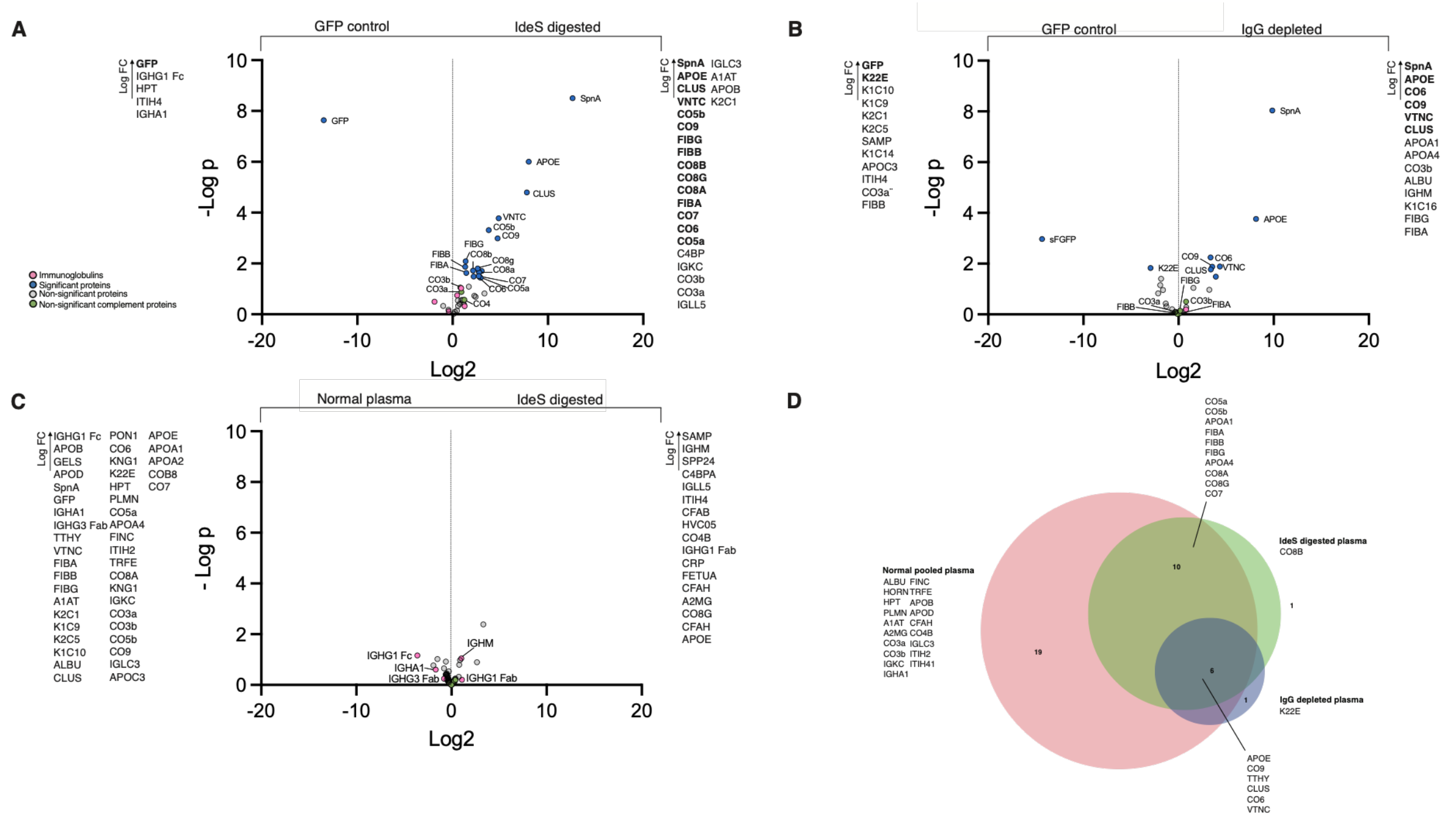

Supplementary figure 5: SpnA human plasma interactome in IdeS-digested and IgG-depleted plasma. Volcano plot comparing the SpnA interactome in human pooled plasma: **A)** digested with IdeS **B)** depleted from IgGs; both with GFP used as control. Significantly enriched proteins are indicated on the sides of the plot in Log2 foldchange (FC) order in bold. **C)** Volcano plot comparing the SpnA interactome in human pooled plasma and IdeS digested plasma. **D)** Venn diagram comparing the significantly enriched human interactors in normal pooled plasma (see Fig. 4), IdeS-digested plasma and IgG-depleted plasma, revealing a core interactome of 6 proteins, including two complement proteins C6 and C9. Whereas C3a, C3b, C5a, C5b, C7, C8α, C8β and C8γ are plasma type specific. The Venn diagram is generated using BioVenn [59]. For AP-MS assays, n = 5 independent pulldowns were performed.

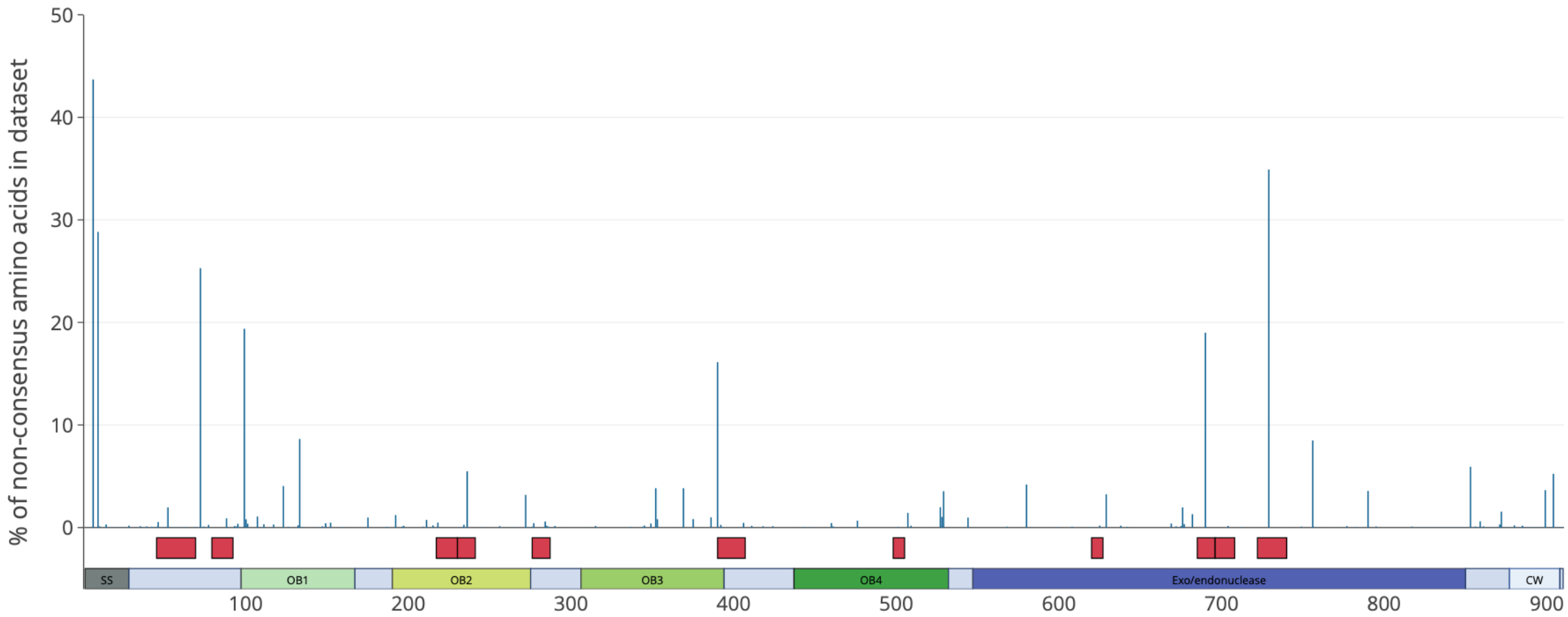

Supplementary figure 6. SpnA sequence variation. Percentage of non-consensus amino acids identified in each residue among 20 580 *S. pyogenes* genomes along the 910 amino acids of the SpnA protein. The regions interacting with the C5b67 complex, as depicted in Fig. 5, are indicated by red boxes below the graph. The different SpnA domains are represented according to the model proposed in this study (Fig. 1B): signal sequence (SS, grey), oligonucleotide-binding domains (OB, shades of green), endo/exonuclease domain (NUCL, dark blue), cell wall attachment LPXTG motif (CW, pale blue).

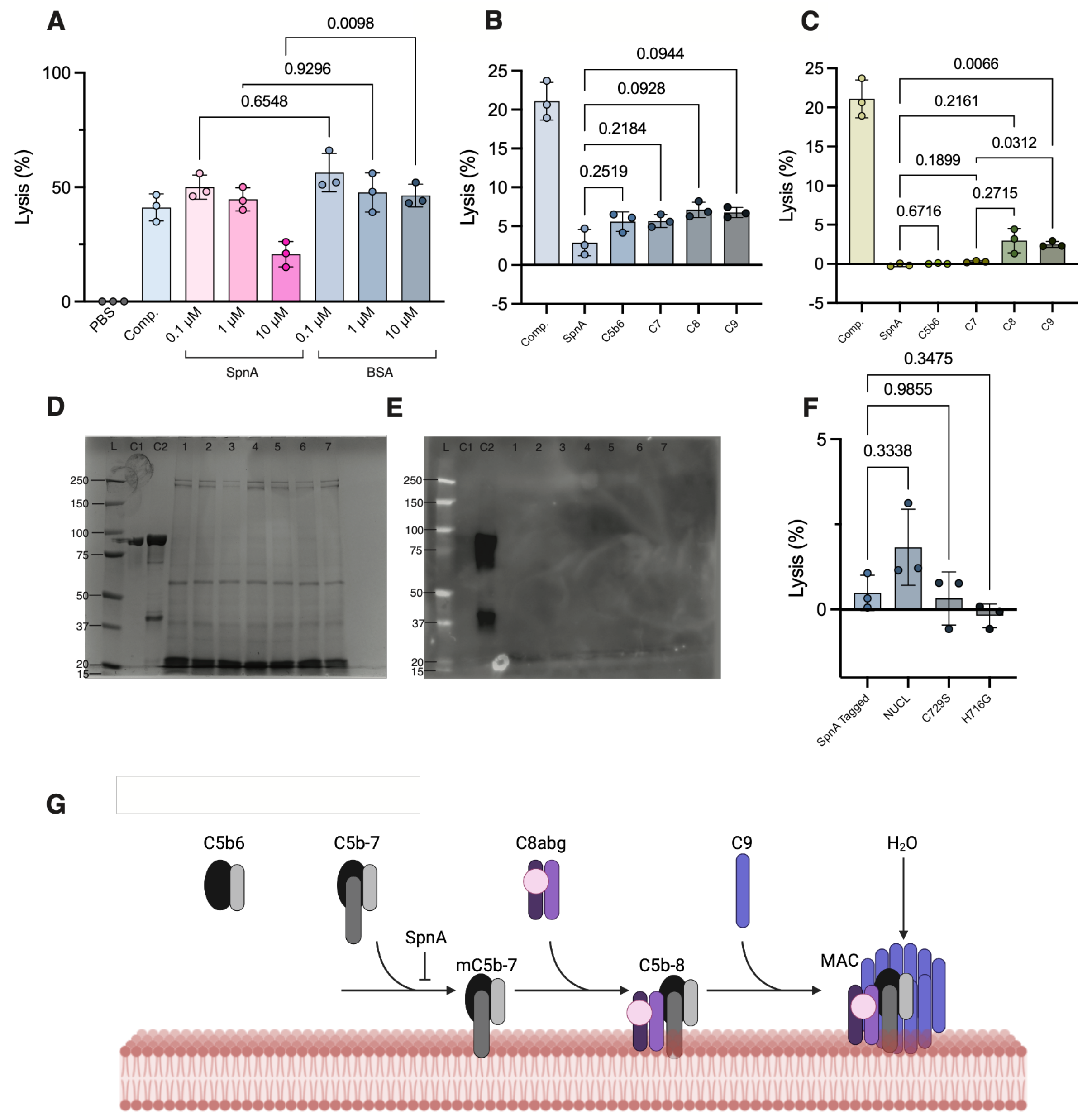

Supplementary figure 7. Hemolysis assay and characterization of SpnA mediated inhibition using human erythrocytes. A) Concentration-dependent hemolysis induced by SpnA compared to bovine serum albumin (BSA) as a negative control. PBS and complement proteins alone were included as controls. Hemolysis was quantified as % lysis relative to controls [16], where 100% hemolysis was obtained by suspending erythrocytes in water. A significant increase in hemolysis was observed for SpnA compared to BSA at 10 µM. Statistical analysis was performed using Brown–Forsythe and Welch one-way ANOVA, exact P-values are stated. **B)** Inhibition of hemolysis using affinity-tagged SpnA. SpnA was added to the samples sequentially at distinct points to assess its' inhibitory effect on hemolysis. Statistical analysis was performed using Brown–Forsythe and Welch one-way ANOVA, as in (A). **C)** Inhibition of hemolytic activity of SpnA mutants. Statistical analysis using Brown–

Forsythe and Welch one-way ANOVA revealed no significant differences. **D**) Representative SDS-PAGE and **E**) western blot analysis of the erythrocyte fraction (pellet after lysis) presented in panel **B**). Affinity-tagged (C2) and untagged (C1) SpnA were included as controls. **F)** Schematic overview of the MAC assembly, illustrating the sequential addition of complement components leading to pore formation in the membrane. The suggested site where SpnA inhibits the MAC assembly is indicated. For the hemolysis assay, n = 3 technical replicates.

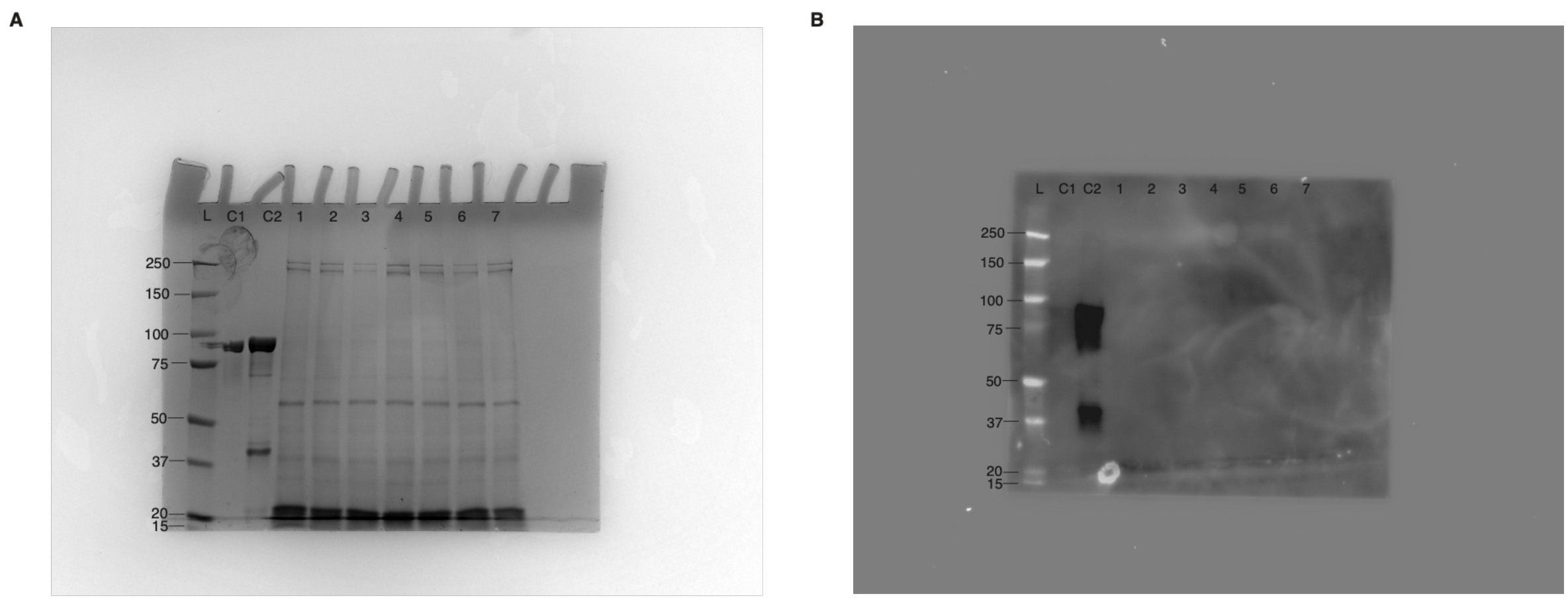

Supplementary figure 8. Uncropped SDS-PAGE gel and western blot of data presented in Supplementary figure 7. A) Uncropped representative SDS-PAGE gel and **B)** western blot from Supplementary figure 7 of the hemolysis assay and characterization of SpnA using human erythrocytes.

Supplementary table 1: Interactions between chain A and chain B in dimeric SpnA mapped by SpotON analysis.

Interactors chain A			Interactors chain B		
Residue Index	Residue Name	Residue Chain	Residue Index	Residue Name	Residue Chain
533	GLY	A	533	GLY	B
534	HIS	A	534	HIS	B
539	LYS	A	539	LYS	B
543	GLN	A	543	GLN	B
544	LYS	A	544	LYS	B
545	ASP	A	545	ASP	B
546	LEU	A	546	LEU	B
572	LYS	A	572	LYS	B
576	GLU	A	576	GLU	B
579	ILE	A	579	ILE	B
580	HIS	A	580	HIS	B
581	ASP	A	581	ASP	B
583	ASN	A	583	ASN	B
619	LYS	A	619	LYS	B
620	LYS	A	620	LYS	B
621	LEU	A	621	LEU	B
622	GLY	A	622	GLY	B

Supplementary table 2: HDX Experimental details and data.

Data Set	apo_SpnA (control)	Mg	Ca	Mg + Ca
HDX reaction details	10 mM PBS, 150 mM NaCl, pH(read)=7.5, pD(read)=7.1, 1:10 Dilution in labelling	10 mM PBS, 150 mM NaCl, 5 mM MgSO4, pH(read)=7.5, pD(read)=7.1, 1:10 Dilution in labelling	10 mM PBS, 150 mM NaCl, 1 mM CaCl2 pH(read)=7.5, pD(read)=7.1, 1:10 Dilution in labelling	10 mM PBS, 150 mM NaCl, 5 mM MgSO4, 1 mM CaCl2, pH(read)=7.5, pD(read)=7.1, 1:10 Dilution in labelling
HDX time course (sec)	30, 300, 3000, 9000	30, 300, 3000, 9000	30, 300, 3000, 9000	30, 300, 3000, 9000
HDX control samples	none	none	none	none
# of peptides	436	436	436	436
Sequence coverage	90.99%	90.99%	90.99%	90.99%
Average peptide length / Redundancy	13.50 / 6.47	13.50 / 6.47	13.50 / 6.47	13.50 / 6.47
Replicates	3	3	3	3
Repeatability (avg. stddev of #D)	0.083	0.0809	0.0747	0.096
Significant differences in HDX (delta HDX > X D - 95% CI)	n/a	0.3451 D	0.3394 D	0.3712 D
