# Supplementary material for: Molecular mechanism of complement interference by Streptococcus pyogenes nuclease A
Source: Commun Biol. 2026 Sep 23;9:1245. doi: 10.1038/s42003-026-10956-9 (PMC13601614; doi:10.1038/s42003-026-10956-9)
Supplement: Supplementary file 2 [file 42003_2026_10956_MOESM5_ESM.pdf]

nature portfolio

Corresponding author(s): Lotta J Happonen

Last updated by author(s): Apr 10, 2026

Reporting Summary

Nature Portfolio wishes to improve the reproducibility of the work that we publish. This form provides structure for consistency and transparency in reporting. For further information on Nature Portfolio policies, see our Editorial Policies and the Editorial Policy Checklist.

Statistics

For all statistical analyses, confirm that the following items are present in the figure legend, table legend, main text, or Methods section.

n/a	Confirmed	
☐	☒	The exact sample size (*n*) for each experimental group/condition, given as a discrete number and unit of measurement
☐	☒	A statement on whether measurements were taken from distinct samples or whether the same sample was measured repeatedly
☐	☒	The statistical test(s) used AND whether they are one- or two-sided<br>*Only common tests should be described solely by name; describe more complex techniques in the Methods section.*
☒	☐	A description of all covariates tested
☐	☒	A description of any assumptions or corrections, such as tests of normality and adjustment for multiple comparisons
☐	☒	A full description of the statistical parameters including central tendency (e.g. means) or other basic estimates (e.g. regression coefficient) AND variation (e.g. standard deviation) or associated estimates of uncertainty (e.g. confidence intervals)
☐	☒	For null hypothesis testing, the test statistic (e.g. *F*, *t*, *r*) with confidence intervals, effect sizes, degrees of freedom and *P* value noted<br>*Give P values as exact values whenever suitable.*
☒	☐	For Bayesian analysis, information on the choice of priors and Markov chain Monte Carlo settings
☒	☐	For hierarchical and complex designs, identification of the appropriate level for tests and full reporting of outcomes
☒	☐	Estimates of effect sizes (e.g. Cohen's *d*, Pearson's *r*), indicating how they were calculated

Our web collection on statistics for biologists contains articles on many of the points above.

Software and code

Policy information about availability of computer code

Data collection	For proteomics experiments,d ata acquisition software packages provided by Thermo Fisher Scientific Inc.<br>For cryoEM data,th e EPU data collection software v3.2.0 was used.
Data analysis	For affinity-purification mass spectrometry we used MaxQuant (v2.6.7.0),P erseus (v2.0.11.0) and GraphPad Prism (vl0.2.3).<br>For cross-linking mass spectrometry we used plink2 (v2.3.11).<br>For hydrogen-deuterium exchange mass spectrometry we used PEAKS Studio X and HDExaminer (v3.4.2).<br>For cryoEM data processing we used cryoSPARC (v.4.6.2),C OOT (v0.9.8.92) and Phenix (vl.21.2-5419-000) and with visualization in pyMOL (v3.1.4.1).<br>For docking we used HADDOCK (v2.5) with analysis using pyMOL (v3.1.3.1) and Python (3.12.9).<br>SpnA allelles and MSA were done in chewBBACA.

For manuscripts utilizing custom algorithms or software that are central to the research but not yet described in published literature, software must be made available to editors and reviewers. We strongly encourage code deposition in a community repository (e.g. GitHub). See the Nature Portfolio guidelines for submitting code & software for further information.

Data

Policy information about availability of data

All manuscripts must include a data availability statement. This statement should provide the following information, where applicable:

- Accession codes, unique identifiers, or web links for publicly available datasets
- A description of any restrictions on data availability
- For clinical datasets or third party data, please ensure that the statement adheres to our policy

Coordinates of the SpnA monomer and dimer structures and the corresponding cryoEM density map have been deposited to the Protein Data Bank under accession codes 9SF8 and 9SF9, respectively. The affinity-pulldown and XL-MS data have been deposited to the ProteomeXchange consortium via the MassIVE partner repository https://massive.ucsd.edu/ with the dataset identifier PXD076782. The HDX-MS data and HDExaminer analysis files have been deposited to the ProteomeXchange Consortium via the MassIVE partner repository with the dataset identifier PXD076781.

Research involving human participants, their data, or biological material

Policy information about studies with human participants or human data. See also policy information about sex, gender (identity/presentation), and sexual orientation and race, ethnicity and racism.

Reporting on sex and gender	No data has been collected on sex or gender.
Reporting on race, ethnicity, or other socially relevant groupings	No data has been collected on race, ethnicity, or other socially relevant groupings.
Population characteristics	No population characteristics were analysed.
Recruitment	Informed consent was obtained prior to blood collection from 3 healthy donors.
Ethics oversight	Lund University

Note that full information on the approval of the study protocol must also be provided in the manuscript.

Field-specific reporting

Please select the one below that is the best fit for your research. If you are not sure, read the appropriate sections before making your selection.

☒ Life sciences ☐ Behavioural & social sciences ☐ Ecological, evolutionary & environmental sciences

For a reference copy of the document with all sections, see nature.com/documents/nr-reporting-summary-flat.pdf

Life sciences study design

All studies must disclose on these points even when the disclosure is negative.

Sample size	Sample size of all experiments were chosen based on common practice in the fled; without any statistical sample size calculations.
Data exclusions	No data were excluded from the analysis.
Replication	Affinity-purification experiments were replicated in live replicates. Cross-linking and hydrogen-deuterium exchange mass spectrometry experiments were performed as duplicates. For hemolysis assays we had three biological replicates. CryoEM data was collected from one grid and the CD spectroscopy data is based on one measurement.
Randomization	This is a molecular biology study, where sample randomization is not relevant.
Blinding	This is a molecular biology study, where binding is not relevant.

Reporting for specific materials, systems and methods

We require information from authors about some types of materials, experimental systems and methods used in many studies. Here, indicate whether each material, system or method listed is relevant to your study. If you are not sure if a list item applies to your research, read the appropriate section before selecting a response.

Materials & experimental systems

n/a	Involved in the study
☒	☐ Antibodies
☒	☐ Eukaryotic cell lines
☒	☐ Palaeontology and archaeology
☒	☐ Animals and other organisms
☒	☐ Clinical data
☒	☐ Dual use research of concern
☒	☐ Plants

Methods

n/a	Involved in the study
☒	☐ ChIP-seq
☒	☐ Flow cytometry
☒	☐ MRI-based neuroimaging

Plants

Seed stocks	No plants were used in this study.
Novel plant genotypes	No plants or novel plant genotypes were used in this study.
Authentication	No authentication was done.
