# Supplementary material for: Molecular mechanism of complement interference by Streptococcus pyogenes nuclease A
Source: Commun Biol. 2026 Sep 23;9:1245. doi: 10.1038/s42003-026-10956-9 (PMC13601614; doi:10.1038/s42003-026-10956-9)
Supplement: Supplementary file 3 [file 42003_2026_10956_MOESM2_ESM.docx]

**Description of Additional Supplementary Files**

File name: Supplementary Data 1

Description: Source data for findings presented in main figures

File name: Supplementary Data 2

Description: The SpnA DNA sequence alleles and their changes in regions interacting with C5b7 complex
